# Using Passive Smartphone Sensing for Improved Risk Stratification of Patients With Depression and Diabetes: Cross-Sectional Observational Study

**DOI:** 10.2196/11041

**Published:** 2019-01-29

**Authors:** Archana Sarda, Suresh Munuswamy, Shubhankar Sarda, Vinod Subramanian

**Affiliations:** 1 Sarda Centre for Diabetes and Selfcare Aurangabad India; 2 DST Health Informatics Rapid Design Lab Indian Institute of Public Health-Hyderabad Hyderabad India; 3 Touchkin eServices Private Limited Bangalore India

**Keywords:** depression, diabetes, mental health, comorbidity, passive sensing, smartphone, classification, machine learning, mHealth, risk assessment

## Abstract

**Background:**

Research studies are establishing the use of smartphone sensing to measure mental well-being. Smartphone sensor information captures behavioral patterns, and its analysis helps reveal well-being changes. Depression in diabetes goes highly underdiagnosed and underreported. The comorbidity has been associated with increased mortality and worse clinical outcomes, including poor glycemic control and self-management. Clinical-only intervention has been found to have a very modest effect on diabetes management among people with depression. Smartphone technologies could play a significant role in complementing comorbid care.

**Objective:**

This study aimed to analyze the association between smartphone-sensing parameters and symptoms of depression and to explore an approach to risk-stratify people with diabetes.

**Methods:**

A cross-sectional observational study *(Project SHADO—Analyzing Social and Health Attributes through Daily Digital Observation)* was conducted on 47 participants with diabetes. The study’s smartphone-sensing app passively collected data regarding activity, mobility, sleep, and communication from each participant. Self-reported symptoms of depression using a validated Patient Health Questionnaire-9 (PHQ-9) were collected once every 2 weeks from all participants. A *descriptive analysis* was performed to understand the representation of the participants. A *univariate analysis* was performed on each derived sensing variable to compare behavioral changes between depression states—*those with self-reported major depression (PHQ-9>9)* and *those with none (PHQ-9≤9)*. A *classification predictive modeling*, using supervised machine-learning methods, was explored using derived sensing variables as input to construct and compare classifiers that could risk-stratify people with diabetes based on symptoms of depression.

**Results:**

A noticeably high prevalence of self-reported depression (30 out of 47 participants, 63%) was found among the participants. Between depression states, a significant difference was found for *average activity rates (daytime)* between participant-day instances with symptoms of major depression (mean 16.06 [SD 14.90]) and those with none (mean 18.79 [SD 16.72]), *P*=.005. For *average number of people called (calls made and received)*, a significant difference was found between participant-day instances with symptoms of major depression (mean 5.08 [SD 3.83]) and those with none (mean 8.59 [SD 7.05]), *P*<.001. These results suggest that participants with diabetes and symptoms of major depression exhibited lower activity through the day and maintained contact with fewer people. Using all the derived sensing variables, the extreme gradient boosting machine-learning classifier provided the best performance with an average cross-validation accuracy of 79.07% (95% CI 74%-84%) and test accuracy of 81.05% to classify symptoms of depression.

**Conclusions:**

Participants with diabetes and self-reported symptoms of major depression were observed to show lower levels of social contact and lower activity levels during the day. Although findings must be reproduced in a broader randomized controlled study, this study shows promise in the use of predictive modeling for early detection of symptoms of depression in people with diabetes using smartphone-sensing information.

## Introduction

### Background

There exists growing evidence regarding the bidirectional association between diabetes (type 2) and depression [1,2]. A meta-analysis of published studies on adults [3] reported depression to be 2 to 3 times more common in people with diabetes (both types) than in those without, with the odds of depression significantly higher in women than in men with diabetes. An estimated 8% to 35% of people across ages with diabetes mellitus (both type 1 and 2) also suffer from depression [4]. Depression increases the risk of nonadherence to medical treatment by 27% to 30% [5-7], which is a significant problem in diabetes self-care. Comorbidity (diabetes and depression) has also been associated with increased health care costs. Individuals (US adults) with diabetes who also had depression were found to be 2 to 4.5 times more expensive to treat than those with diabetes alone (US $247 million compared with US $55 million in 2001, dollars after adjusting for differences in age, sex, race, ethnicity, health insurance, and comorbidity) [8,9]. Depression is known to be associated with abnormalities in metabolism of biologics (eg, increased counterregulatory hormone release and action, changes in glucose transport function, and increased immuno-inflammatory activation) [10]. Depression might also increase the risk of developing type 2 diabetes with an increase in insulin resistance and reduction of glucose uptake in adults [11]. Comorbidity of depression and diabetes is associated with a high likelihood of complications [12,13], lower quality of life [14], increased mortality [15], poor management and control [16,17], and poor disease outcomes through decreased physical activity [18,19] as reported in studies covering a diverse population across age groups.

In an analysis of worldwide studies [20], it was found that primary care physicians fail to correctly diagnose between 30% and 50% of patients who present with a depressive disorder. The same study also pointed to poor recognition rates of depression symptoms among both men and women aged less than 40 years. A retrospective study [21] among a population- based sample of primary care patients with diabetes (both type 1 and type 2, across age groups) within a US-based health maintenance organization revealed that depression is identified only half of the time (approximately 51%). The same study also pointed that only 31% of patients with comorbid diabetes and depression received adequate antidepressant treatment and only 6.7% received 4 or more psychotherapy sessions during a 12-month period. Clinical-only interventions seem to have very modest effects in diabetes management of patients with depression [22-24]. The American Diabetes Association recommends that patients with diabetes be screened for psychosocial and psychological problems or disorders, such as depression [4,25]. However, this appears to happen rarely [26].

The number of global smartphone users is expected to surpass 2.3 billion by 2017 [27]. Smartphones carry sensors such as accelerometer, global positioning system (GPS), and ambient light sensors that capture data and that could provide information on someone’s behavior. In this context, the smartphone could be the most ubiquitous data collection device today. It also presents with huge privacy and security concerns. Developed nations have higher smartphone penetration, and the ownership rates in emerging and developing nations have been rising at an extraordinary rate [28]. Passive data from smartphone sensors have been known to detect patterns of behavior in people with depression [11,29-32]. Research has been establishing the link between smartphone-sensing data and its application in overall well-being [33-36] and depression [37-39]. Smartphones for social sensing [40,41], in monitoring and possibly as an intervention in mental health [42,43], has the advantage of ubiquity, discretion, and low cost. Comorbidity poses a large economic burden; hence, there exists a need for effective screening and treatment. To use limited resources efficiently, risk stratification is important to target appropriate intervention for people with diabetes and depression.

### Previous Work

Many studies have explored how smartphone-sensing data can be used as a predictor for depression and mental health [44-47], but very few studies have applied passive sensing to predict symptoms of depression among people with diabetes, thereby enabling improved risk stratification. There are recent studies that have attempted to predict depression among patients with diabetes using longitudinal patient records or data from clinical trials or surveys, but not using sensing data as an indicator [48-50].

### Study Objective

The aim of the study was (1) to identify behaviors derived from smartphone-sensing data that are significant with symptoms of major depression compared with those with no symptoms among patients with diabetes and (2) to evaluate a risk stratification approach for early detection of symptoms of depression in patients with diabetes using smartphone-sensing parameters. To the best of our knowledge, this study was the first such implementation of using automatically captured smartphone- sensing data such as activity, communication, mobility, and sleep to screen for symptoms of depression among primary care patients with diabetes.

## Methods

### About the Study

The pilot study named *Project SHADO (Analyzing Social and Health Attributes through Daily Digital Observation)* was conducted in 2016 on a cross section of participants with diabetes located in periurban India and owning low-cost smartphones. The institutional Ethics Committee at the Public Health Foundation of India’s Indian Institute of Public Health at Hyderabad, India, approved the study (Approval number IIPHH/TRCIEC/073/2016).

### Study Design

The study design was conducted in association with a diabetes clinic situated in Aurangabad, a city in the state of Maharashtra, India. A cross-sectional observational study was designed to be conducted on a sample of patients undergoing diabetes treatment at the clinic. The study did not require any intervention or change in treatment or lifestyle for the participants. It did not involve a control group.

The period of the study was originally 14 weeks and later extended to 20 weeks to collect sufficient smartphone-sensing data. The study app that was used passively and anonymously collected data regarding activity, mobility, sleep, and communication from each participant. The actual conversation from the call was never collected. For identifying symptoms of depression among enrolled participants, a globally validated screening tool, Patient Health Questionnaire-9 (PHQ-9) was used [51,52]. The PHQ-9 survey was made available in both English and the local language (Marathi). The language- modified version of PHQ-9 had been validated in other studies [53,54]. The PHQ-9 English and Marathi language questionnaire are available in Multimedia Appendices 1 and 2, respectively. For the participants to feel comfortable keeping data services enabled on their smartphones, they were provided with a 1 GB data recharge per month to cover for usage costs. No other incentive was provided to the participants. The care providers at the clinic administered the study. Administrator guidelines were set that ensured effective participant enrollment and onboarding process. A Web-based patient administration system was used to manage participant details. The clinical staff were oriented about the study, familiarized about the study app, and trained on the administrator system and guidelines. Effective monitoring and support was established to manage any issues that could occur during the study. The conceptual framework of the research design is shown in Multimedia Appendix 3. The study was designed to have no financial burden on the participant, nor any drug or device hazard.

### Study Participants

A list of 100 periurban patients undergoing treatment for diabetes and who satisfied the study inclusion criteria (see Textbox 1) were contacted for their interest and participation in the study. Patients who did not meet the inclusion criteria, who had mobility restrictions, who were bedridden, or who had serious comorbid conditions including disabilities and visual or hearing impairment were excluded.

Overall, 47 out of the 100 patients provided their consent and were enrolled for the study. Participation in the study was voluntary, and as per the informed consent provided, a participant could decide not to participate or could withdraw from the study at any time without having to provide any reasons or justifications. All study participants followed a formal onboarding process where they were provided with information about the study; were educated about the privacy, security, and consent process; had the study app setup; provided explicit consent; and completed the initial PHQ-9 survey. The first self-reported depression score was assessed in person at the clinic followed by collection over telephone once every 2 weeks during the study period.

### Study App

The study used a smartphone-sensing app (“app”) developed by Touchkin. The app assisted family members to care for their loved ones remotely and nonintrusively and to check on their well-being. The app’s machine learning (ML) platform helped detect probable well-being changes by using activity rates, communication levels, sleep patterns, and mobility information collected from the user’s smartphone sensors.

### Data Collection

Data were deidentified before use for research purposes.

#### Sociodemographic Data

The participant’s sociodemographic information such as gender, marital status, occupation, age, education, and family particulars were captured at enrollment. The level of control over the existing diabetes condition for each participant was assessed by the diabetologist. The participant’s level of control was assessed based on their existing condition, lifestyle, and medication adherence history. Participants were classified as having low, moderate, or high control over their condition.

#### Passive Sensing Data

Smartphone-sensing data were captured by the app automatically every 2 minutes and stored on-device using a read or write memory card. The app was designed to capture only hashed identifiers, and the collected data were secured and anonymized on-device before being transferred to the storage servers for an aggregate analysis. All transmissions were in encrypted form using the HTTPS secure sockets layer protocol. On the server side, these files were merged, parsed, and synchronized by Python-based postprocessing infrastructure and stored in not only SQL–based servers. The servers and the data thereof were access restricted, allowing only the engineering lead to retrieve the minimal needed data for research. The raw passive sensing data were processed and daily values derived for the sensing variables. Running of the ML models on the entire deidentified dataset was performed securely on the cloud with the research analyst getting to view only the performance results.

**Study inclusion and exclusion criteria.**

**Inclusion criteria:**

- Those who were 1+ year on diabetes treatment, with recent 6 months of consulting the diabetologist
- Those who had at least one clinic visit per month
- Those who were 18 years and above
- Those who owned a smartphone with Android operating system
- Those with normal mobility, with no known debilitating comorbidities
- Those willing to participate for the duration of the study
- Those willing to carry a smartphone at all times
- Those willing to download the smartphone-sensing app

**Exclusion criteria:**

- Those with known debilitating comorbidities
- Those with known mobility restrictions
- Those who were bedridden
- Those with disabilities
- Those with visual or hearing impairment
- Those who owned a non-Android smartphone (eg, iOS)

The social interaction data were captured from 3 main sensors and the call logs. These were the accelerometer, the GPS, and the ambient light sensor. All these sensors reflect pairwise communication and face-to-face proximity, intensity and nature of social ties, the dynamics of network, and amount of light in the background. To ensure that no loss of sensing data occurred in the event of network drops, they were stored in the participant’s smartphone for up to 3 days.

**Figure 1:**
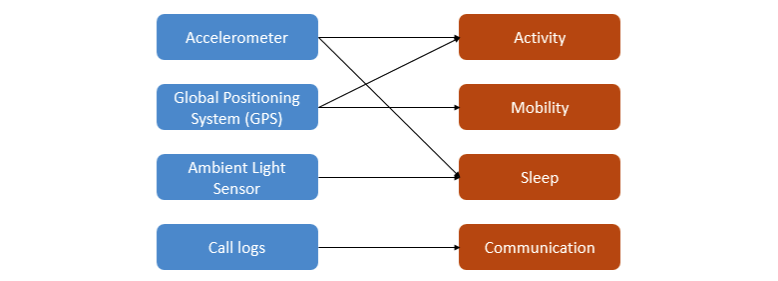
Sensor-feature map.

A total of 53 sensing variables were derived from the activity, mobility, sleep, and communication data collected from the smartphone sensors. Figure 1 outlines the sensor-feature map. Activity variables were derived based on periods where the participant was found to be active. This was measured by calculating the number of times the relative gravity values, derived from accelerometer readings, exceeded the stationary threshold range (as defined from 0.8 to 1.2). Relative gravity is a measure of acceleration experienced by the mobile device with reference to earth’s gravity. Mobility variables were derived based on the number of locations and the distance traveled (in meters). Sleep variables were based on relative gravity and the number of screen-ons. Call-related variables were based on the number of total calls (made and received), missed calls, and call duration (in minutes). Call-related variables do not include participant texting, as this was not considered in the scope of data capture. The values for these variables were derived as day-wise aggregates from the raw-sensing data collected for each participant. Details on each of the 53 derived sensing variables can be found in Multimedia Appendix 4.

#### Screening Instrument Data

PHQ-9 consists of a validated 9-item depression screening tool, with each item having 4 options (scored 0-3) as responses namely: “not at all,” “several days,” “more than half the days,” and “nearly every day.” PHQ-9 helps screen for the presence and severity of depression with a maximum total score of 27. The values for each of the 53 derived sensing variables were aggregated day wise, whereas the screening data (PHQ-9 scores) were collected 14 days apart for each participant. Therefore, it was decided to impute the average of 2 consecutive PHQ-9 surveys submitted by a participant as the score for all the participant-day instances occurring between the 2 screening dates for that participant.

#### Data Quality

Of the 47 participants, only 1 participant did not complete the study. The final analysis was performed on data from 46 participants, of which 29 were men and 17 were women. Over the course of the study, a total of 2694 *participant-day* records (instances) were collected. This formed the dataset for subsequent analysis.

All PHQ-9 surveys had reminders for the participants as well as administrators to follow up. The opportunity to miss capture of sensing data could arise from many reasons, and mostly these are random in nature. Some include participants changing phone settings, forgetting to carry phones or there are errors during capture, storage, and manipulation. This resulted in dataset instances having values missing for some of the derived sensing variables. Data quality for smartphone-sensing data was an area of focus during the data gathering and data cleaning phase. Typically, low-end smartphone devices present several challenges when sending out passive sensing data, namely, frequency of data transmission varies, unexpected shutdown occurs for extended periods during the day, and data values (eg, accelerometer readings) sometimes do not match higher-end devices. Within the study, data quality measures were implemented to solve 2 issues: (1) data integrity: how to conduct reliable sampling of data to ensure there was minimum loss in continuity of data feeds and (2) data accuracy: how to ensure the validity of the data being collected, so that we could be confident that this correctly represented participant context (see Multimedia Appendix 5 for the measures taken to ensure data integrity and accuracy).

### Monitoring and Support

During the study period, active support was provided to ensure minimal dropout and to ensure priority resolution of issues and monitoring of data quality. The administrators monitored the Web administration system for alerts and reached out to participants as required. Alerts included unusual smartphone usage or when 2-week surveys were due and other reasons (eg, not receiving sensing data).

#### Smartphone Battery and Memory Optimization

The app’s technical and proprietary data collection methods ensured that the participant’s smartphone battery impact was kept low. The app occupied less than 10 MB of storage space on a typical Android smartphone and consumed less than 2% of total battery. This was lower than that consumed by other apps usually installed on a smartphone and as measured over a 24-hour period. A recent study [55] had pointed out the importance of reporting battery performance as it plays a major role in sensing data collection and quality.

### Data Analysis

Analysis was performed in 3 parts: descriptive, univariate, and classification modeling.

*Descriptive analysis* was performed to understand the representation of the participants based on their sociodemographic factors, clinical presentations, and mental well-being.

*Univariate analysis* was performed to understand whether there were observable differences in behavior between a set of instances tagged with symptoms of major depression (depressed—D) and those with none (not depressed—ND). The D class included instances with PHQ-9 greater than 9 (moderate to severe severity) scores, and the ND class included instances with PHQ-9 less than or equal to 9 (none to mild severity) scores. The PHQ-9 cutoff for major depression was decided based on published studies [56]. An independent *t* test was conducted to compare the 2 classes, considering the test’s robustness with large number of instances [57]. Therefore, it was safe to assume that the data in each class were normally distributed for each derived sensing variable. It was also observed that the number of instances in the 2 classes for each derived sensing variable was significantly unbalanced. It was, therefore, decided that the unequal variance independent *t* test [58] be applied (with and without outliers) to compare the 2 classes. It was observed that any imputation of missing values in sensing variables would potentially introduce bias, and hence no treatment was affected on the missing values.

*Classification modeling* was performed with the objective to explore, compare, and identify the best performing classifier method to build a risk stratification model for early detection of symptoms of depression in participants with diabetes. A total of 5 supervised ML methods and their ensemble were explored. Tree-based supervised ML methods were mostly considered, given their robustness to multicollinearity, outliers, and missing values. These include support vector machine (SVM), decision tree (DT), random forest (RF), adaptive boosting (AdaBoost), and extreme gradient boosting (XGBoost). The radial basis function (RBF) kernel used in SVM methods is known to handle large feature sets and their nonlinear interactions. The other 4 methods were tree-based methods that included the basic DT along with boosting trees (AdaBoost and XGBoost) and bagging trees (RF). Both bagging and boosting trees combine several DTs to reduce error and improve classification performance. Boosting trees help reduce bias, whereas bagging trees help reduce variance. A voting ensemble was also trained that combined each of the 5 methods to check for improved classification performance. Each of the 5 methods provides a class (D or ND) prediction (vote) for each participant-day instance, whereas the voting ensemble counts these votes and tags the majority class voted for that instance.

**Figure 2:**
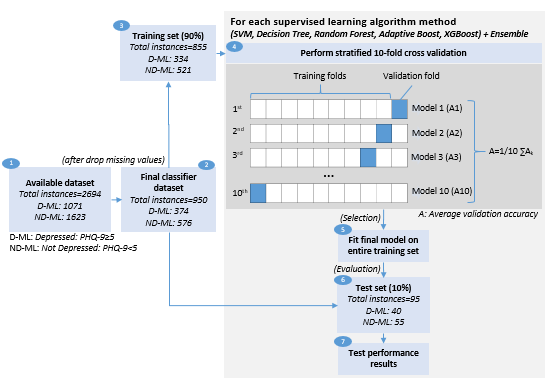
Classification modeling (train-validate-test) approach. PHQ-9: Patient Health Questionnaire-9; SVM: support vector machine; XGBoost: extreme gradient boosting.

Open-source Python software on Jupyter Notebook was used for modeling. A lower PHQ-9 cutoff of 5 was considered for the modeling to broaden the scope and include instances with mild symptoms of depression.

Mild symptoms or subclinical depression in people with diabetes has been found to be common, associated with high levels of diabetes-related distress, psychological distress, and lower quality of life and also a risk indicator of major depression [59-61]. Those instances with self-reported symptoms of depression (PHQ-9≥5—mild to severe) were grouped under the depressed-machine learning (D-ML) class and those with none (PHQ-9<5) under the not depressed-machine learning (ND-ML) class. Instances that contained missing values in any of the 53 derived sensing variables were entirely dropped. The dataset so obtained was divided into a training set (90%) and test set (10%), and a stratified K-fold cross-validation (CV) with K equal to 10 folds was performed on the training set for each of the 5 classifier methods (see Figure 2 for the modeling approach [train-validate-test] followed). The CV has been widely used to compare different classifier methods. A major advantage of using the 10-fold CV approach is that every data instance gets to be in a validation set exactly once and gets to be in a training set 9 times, leading to lower variance in the resulting estimate. Stratification ensures that the ratio of both classes (D-ML and ND-ML) is equally represented in each fold. A CV splits the training set into K equal parts. A model is trained on K−1 parts and gets validated on the remaining part. This process leads to development of K models for each method. The average performance of K models is then compared for each method. A nested CV was not opted for because of computational challenges. The final model for each method was then trained on the entire training set and tested with the unseen test set. Test performance results were then compared to identify the best ML method. Classification performance in terms of accuracy, specificity, sensitivity, and precision along with the confusion matrix was used to compare the ML methods. For this study, a lower number of false negatives (wrongly classifies symptoms of depression to be absent) was important as a wrong classification would lead to patients with symptoms of depression being missed out for priority diabetes care. Therefore, apart from high accuracy, a high recall with a reasonably high precision formed the basis to compare and select the appropriate method to build risk stratification models.

## Results

### Descriptive Analysis

**Figure 3:**
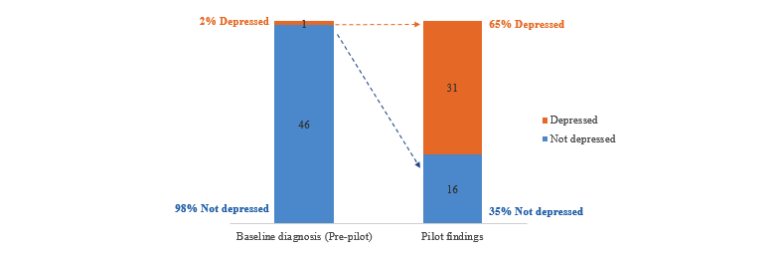
Prevalence of depression.

At the start of the study, 1 out of the 47 participants had a known diagnosis for depression. A noticeably high percentage of participants (30/46), excluding 1 with a known diagnosis, self-reported symptoms of depression on the PHQ-9 survey during the study (Figure 3). Overall, 6 out of 31 reported severe symptoms of depression, including suicidal tendency. These severe cases were referred to psychologists promptly. The diabetologist ensured regular follow-up of their referred patients for their depression condition as per the care protocol established at the diabetes clinic.

At the beginning, 30 men and 17 women participated in the study. Of them, 1 male participant dropped out making it 29 men and 17 women at the end of study. As seen in Table 1, participants had a mean age of 35 years (SD 12). In total, 60% (28/46) of the participants were in the age group of 21 to 40. Of them, 58% (27/46) of the participants were married. A majority of the participants were office goers at 69% (32/46), whereas 17% (8/46) were students. Of them, 58% (27/46) of the participants held a bachelor’s or master’s degree, whereas 34% (16/46) had completed schooling. Some of the clinical characteristics of the participants included an almost equal mix of diabetes condition (type 1/type 2) and 63% (29/46) had moderate level of control over their diabetes condition.

### Univariate Analysis

All the 2694 instances (participant-day records) were included for this analysis. The results with and without outliers are summarized in Tables 2 and 3.

- A significant difference was observed in the *average activity rates in the morning hours (from 6:00 am until 11:59 am)* among those with symptoms of major depression (mean 13.70 [SD 14.04]) compared with those with none (mean 18.48 [SD 18.44]), *P*<.001. A significant difference was also observed in *average activity rates in the remaining part of the day (from noon until 4:00 pm)* among those with symptoms of major depression (mean 16.06 [SD 14.90]) than those with none (mean 18.79 [SD 16.72]), *P*=.005. These results suggested that those with symptoms of major depression exhibited lower and irregular activity rates through the day as compared with those with none.
- A significant difference was observed in the *number of screen-on times at night (from midnight until 6:00 am)* among those with symptoms of major depression (mean 6.70 [SD 9.33]) compared with those with none (mean 3.16 [SD 8.91]), *P*<.001. The results suggested that those with symptoms of major depression possibly had an impacted sleep quality due to higher screen-ons.
- A significant difference was observed in the *average total number of calls (made and received)* among those with symptoms of major depression (mean 12.61 [SD 9.15]) compared with those with none (mean 22.28 [SD 50.76]), *P*<.001. A significant difference was also observed in the *average number of people called* among those with symptoms of major depression (mean 5.08 [SD 3.83]) compared with those with none (mean 8.59 [SD 7.05]), *P*<.001. The results suggested that those with symptoms of major depression maintained contact with fewer people and attended fewer calls.
- Mobility variables showed limited to no statistical significance at 95% and 99% CI, respectively, between the 2 depression states and hence have not been reported.

**Figure 4:**
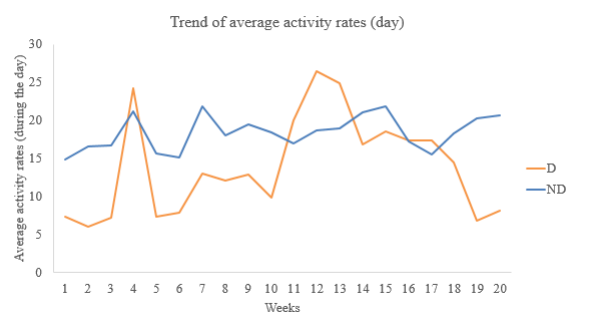
Week-wise trend of average activity rates (day). D: depressed; ND: not depressed.

Univariate trends over the weeks also showed that those with symptoms of major depression (D) exhibited irregular and lower daytime average activity rates (Figure 4) compared with those with none (ND).

**Figure 5:**
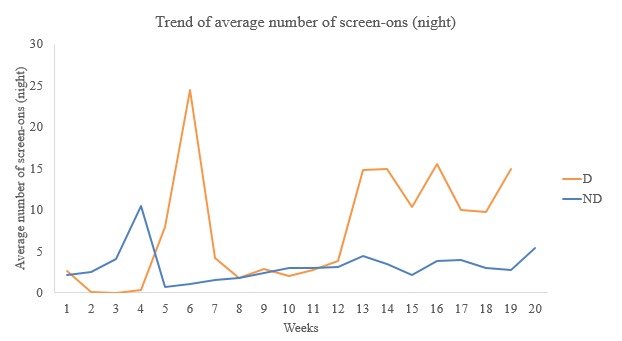
Week-wise trend of average screen-ons (night). D: depressed; ND: not depressed.

Trends over the week showed that those with symptoms of major depression (D) had irregular and higher average number of screen-ons at nighttime (Figure 5) than those with none (ND).

**Figure 6:**
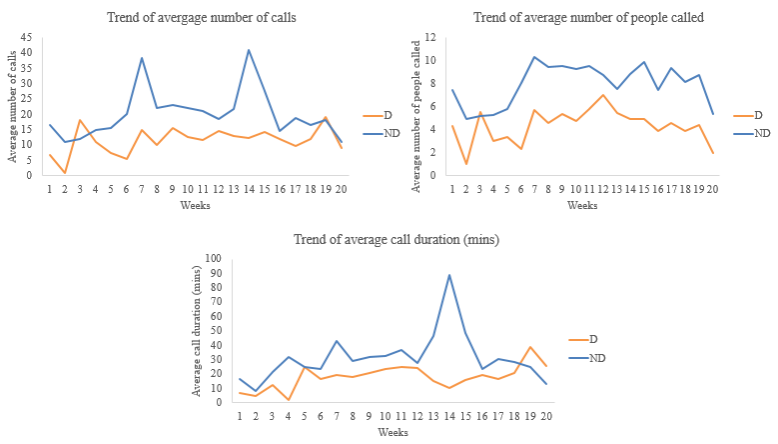
Week-wise trends of average calls-people-duration. D: depressed; ND: not depressed.

Trends over the week also showed that those with symptoms of major depression (D) withdrew socially with lower average number of calls, lower average number of people contacted, and lower average duration per call (Figure 6) than those with none (ND).

**Table 1:** Participant demographics (N=46).

Participant characteristics		Statistic, n (%)
**Age (years)**		
	15-20	4 (10)
	21-30	14 (30)
	31-40	15 (32)
	41-50	5 (11)
	51+	8 (17)
**Gender**		
	Men	29 (64)
	Women	17 (36)
**Marital status**		
	Single	18 (40)
	Married	28 (60)
**Education**		
	Grade 10-12	15 (34)
	Bachelor’s degree	19 (41)
	Master’s degree	9 (19)
	Vocational education	3 (6)
**Occupation**		
	Student	8 (17)
	Home	6 (13)
	Office	32 (70)
**Family**		
	Living alone	4 (9)
	Living with family	42 (91)
**Chronic condition**		
	Diabetes type 1	21 (45)
	Diabetes type 2	25 (55)
**Patient location**		
	Outstation	14 (30)
	In city	32 (70)
**Level of control over diabetes condition**		
	Low	11 (23)
	High	7 (15)
	Moderate	28 (62)

**Table 2:** Univariate analysis results (with outliers).

Key smartphone-sensing variables	Depressed (PHQ-9^a^>9)		Not depressed (PHQ-9≤9)		*P* value (with unequal variance)
	n1^b^ (%)	Mean (SD)	n2^c^ (%)	Mean (SD)	
Activity rate^d^ (am^e^)	194 (11)	13.70 (14.04)	1598 (89)	18.48 (18.44)	<.001
Activity rate (day^f^)	228 (12)	16.06 (14.91)	1761 (88)	18.79 (16.72)	.005
Screen-on^g^ (night^h^)	130 (9)	6.70 (9.33)	1301(91)	3.16 (8.91)	<.001
Calls (made and received)	262 (11)	12.61 (9.15)	2057 (89)	22.28 (50.76)	<.001
People called	262 (11)	5.08 (3.83)	2057 (89)	8.59 (7.05)	<.001
Call duration (minutes)	262 (11)	18.95 (19.32)	2057 (89)	37.59 (174.88)	<.001

^a^PHQ: Patient Health Questionnaire.

^b^n1: Number of instances with values for depressed.

^c^n2: Number of instances with values for not depressed.

^d^Total number of *active* polled every 2 min. *Active:* where relative gravity values exceed the stationary threshold range (0.8-1.2).

^e^From 6:00 am until 11:59 am.

^f^From noon until 4:00 pm.

^g^Total number of *Screen-on* polled every 2 min. *Screen on:* where the user had their mobile screen switched on and unlocked.

^h^From midnight until 6:00 am.

**Table 3:** Univariate analysis results (without outliers).

Key smartphone-sensing variables	Depressed (PHQ^a^-9>9)		Not depressed (PHQ-9≤9)		*P* value (with unequal variance)
	n1^b^ (%)	Mean (SD)	n2^c^ (%)	Mean (SD)	
Activity rate^d^ (am^e^)	183 (11)	11.06 (7.93)	1507 (89)	14.87 (10.80)	<.001
Activity rate (day^f^)	214 (11)	12.95 (6.96)	1754 (89)	18.61 (16.49)	<.001
Screen-on^g^ (night^h^)	120 (9)	4.58 (5.54)	1156 (91)	1.32 (1.69)	<.001
Calls (made and received)	254 (12)	11.69 (7.58)	1933 (88)	16.02 (11.54)	<.001
People called	240 (11)	4.22 (2.37)	1965 (89)	7.59 (5.34)	<.001
Call duration (minutes)	246 (11)	15.24 (12.52)	1917 (89)	21.36 (19.21)	<.001

^a^PHQ: Patient Health Questionnaire.

^b^n1: Number of instances with values for depressed.

^c^n2: Number of instances with values for not depressed.

^d^Total number of *active* polled every 2 min. *Active:* where relative gravity values exceed the stationary threshold range (0.8-1.2).

^e^From 6:00 am until 11:59 am.

^f^From noon until 4:00 pm.

^g^Total number of *Screen-on* polled every 2 min. *Screen on:* where the user had their mobile screen switched on and unlocked.

^h^From midnight until 6:00 am.

### Classification Analysis

All participant-day instances with missing values in 1 or more of the 53 derived sensing variables were removed, which resulted in 950 out of 2694 instances available for analysis. A 90:10 (training:test) split resulted in 855 instances in the training set and 95 instances in the test set. A stratified 10-fold CV was performed on this training set before testing on an unseen test set.

#### Collinearity Check

Very low correlation (*r* values ranging from −.15 to .13) was observed between the self-reported depression state *(PHQ-9 cutoff 5)* and each of the 53 derived sensing variables as measured by Pearson correlation. High pair-wise correlation or collinearity (>80%) was observed among some of the derived sensing variables (Multimedia Appendix 6). Mobility and communication-based variables showed higher collinearity. This was primarily due to the association between the variables and their subset, for example, “total calls at peak” and “total calls at off-peak” variables were subsets of the “total calls” variable. Activity and sleep-based variables showed lower collinearity. This was again due to the association between variables such as “total activity rates,” “screen-on times,” and their “time of day” subsets. For example, “total activity by day/night/eve/am” were all subsets of the “total activity” variable. As each derived sensing variable, of its own, provided rich behavioral context, it was decided to retain all the 53 variables and their subsets as inputs into the modeling.

#### Model Development

In total, 5 ML methods (SVM, DT, RF, AdaBoost, and XGBoost) and their ensemble were trained and compared for performance. Accuracy provided the fraction of correctly classified samples of both classes (D-ML and ND-ML). XGBoost and RF performed the best in terms of accuracy. They reported an average cross-validated accuracy of 79.1% (95% CI 74% to 84%) and 78.3% (95% CI, 71% to 85%), respectively, and a higher test accuracy of 81.1% and 80.0%, respectively (see Table 4) *.* Both the methods also reported a higher recall of 75.0% and 70.0%, respectively, and a reasonable precision of 78.9% and 80.0%, respectively, as compared with other methods. Recall can be interpreted as “Of the participant-day instances that were actually symptoms of depression, what proportion was classified as having symptoms of depression.” Precision can be interpreted as “Of the participant-day instances that were classified as symptoms of depression, what proportion actually had symptoms of depression.”

**Table 4:** Classification performance.

Performance		SVM^a^ (RBF^b^)	Decision tree – single	Random forest	Extreme gradient boosting	Adaptive boost	Voting ensemble
**Accuracy**							
	Average cross-validation accuracy, % (95% CI)	73.8 (67-81)	69.1 (60-78)	78.3 (71-85)	79.1 (74-84)	74.3 (67-81)	75.3 (68-82)
	Test accuracy, %	80.0	66.3	80.0	81.1	73.7	77.9
	Precision (test), %	86.2	60.5	80.0	78.9	75.9	80.6
	Sensitivity and recall (test), %	62.5	57.5	70.0	75.0	55.0	62.5
	Specificity (test), %	92.7	72.7	87.3	85.5	87.3	89.1
**Confusion matrix: training counts**							
	True positive	228	304	324	330	241	321
	True negative	487	503	521	520	485	519
	False positive	34	18	0	1	36	2
	False negative	106	30	10	4	93	13
**Confusion matrix: test counts**							
	True positive	25	23	28	30	22	25
	True negative	51	40	48	47	48	49
	False positive	4	15	7	8	7	6
	False negative	15	17	12	10	18	15

^a^SVM: support vector machine.

^b^RBF: radial basis function.

## Discussion

### Principal Findings

A noticeably high prevalence of self-reported symptoms of depression (63%) was observed in this study as compared with the 8% to 35% normally reported in other studies [4]. This could be attributable to the single study site and the characteristics of the recruited participants. A detailed analysis was not within the scope of study.

Low correlation was observed between self-reported symptoms of depression and each of the derived sensing variables. This contrasts from the results observed in the Dartmouth Student Life study [41] where results indicated a strong correlation between automatic sensing data (derived for sleep, conversation, and location) and PHQ-9 scores. The difference can be attributed to the use of different sets of derived sensing variables and possibly due to missing values in the sensing variables. Again, a detailed analysis on correlation was not within the scope of study. The study did show, at 95% and 99% significance, lower levels of social contact (total calls—made and received), higher phone access at night (number of screen-ons), and lower daytime activity (activity rates during the day) among those with self-reported symptoms of depression (PHQ-9>9).

In total, 1744 out of 2694 participant-day instances were removed from the dataset as they contained missing values in 1 or more of the derived sensing variables. Between large numbers of training instances available for modeling and avoidance of any bias being introduced in the dataset because of imputation of missing values in the derived sensing variables, a decision was taken in favor of the latter. The XGBoost and RF methods were able to classify each participant-day instance with a test accuracy of 81.1% and 80.0%, respectively, and with a sensitivity and recall of 75.0% and 70.0%, respectively. From among the recently published passive sensing studies, only 1 study [38] was found comparable with the approach followed in our study. In that, they used a smartphone app to collect sensing data, used a PHQ-9 self-report scale but with a cutoff of 11 to separate participants into 2 classes, and also built binary classifiers with a leave-one-out CV approach to predict symptoms of depression. That study used 2 classification methods, namely RF and SVM, which resulted in an accuracy of 61% and 59%, respectively, and a sensitivity and recall of 62% and 72%, respectively. Although the results obtained from the *Project SHADO* study did show better performance, it would not be appropriate to make a direct comparison given the different study design adopted by both papers. However, both studies did show a performance superior to a random classification. The classifier results show promise and points to a need to develop high-performing ML models, including evaluation of unsupervised learning approaches as ML and smartphone-sensing technologies advance. A high classifier performance allows for an early and improved detection of symptoms of depression among patients with diabetes. This enables the primary care physician to use the results of the classifier as one of the several biomarkers for high-risk classification and prioritization of patients with diabetes and provide for personalized and empathetic care.

### Limitations

Key limitations of the study included a single study site, small participant size, and the nonrandomized–based approach. The average of 2 consecutive PHQ-9 scores reported by a participant was assumed to be the depression symptom of the participant for the days and instances between the 2 screening time points. This could induce an error in outcome on days or instances where a participant was to exhibit a different mood or symptom. There is a need to investigate a better approach to capture daily symptoms of depression for each participant instead of the imputation approach taken for this study. PHQ-9, although a validated scale to screen for symptoms of depression, is not a tool to firmly diagnose depression [62]. Therefore, participants with high PHQ-9 scores need not necessarily have depression and vice versa. The study was also limited by missing values in derived sensing variables. The study design should include a plan for specific follow-up with participants, without influencing the participants, to help reduce possibilities of missing values in sensing information that might be introduced due to the participant’s smartphone usage behavior. Although several measures to monitor and ensure data quality were used, limitations do exist in the data collection methods. Data collection methods can be improved further to manage the variability of mobile devices and how they respond to the data collection code set. The approach and observations from this pilot study are at best preliminary and a larger, randomized control–based study would help in validation of the findings.

### Conclusions

Smartphone sensor–enabled daily digital observation of health and social attributes is a promising new approach with significant potential for management of comorbid conditions. Although the findings need to be replicated with a larger multisite randomized control study, this observational study has opened up the possibility of understanding the real-world everyday mental well-being and social attributes of people with diabetes in a clinical setting. Supplementing the smartphone- sensing data with clinical records from each visit along with daily behavioral information aggregated from a smartphone-based conversational chatbot app [63] would help further the risk stratification objectives. It is equally important to be sensitive and treat passive sensing data as sensitive health information and ensure adequate privacy and security controls are in place before wider use.

## Appendix

Multimedia Appendix 1 Patient Health Questionnaire- 9 English language version.

Multimedia Appendix 2 Patient Health Questionnaire- 9 Marathi language version.

Multimedia Appendix 3 Conceptual framework of study research design.

Multimedia Appendix 4 Derived smartphone-sensing variable description.

Multimedia Appendix 5 Measures taken to ensure sensing data integrity and accuracy.

Multimedia Appendix 6 Pearson correlation of derived sensing variables and depression state.

## Abbreviations

AdaBoost: adaptive boosting
CV: cross-validation
D: depressed
D-ML: depressed-machine learning
DT: decision tree
GPS: global positioning system
ML: machine learning
ND: not depressed
ND-ML: not depressed-machine learning
PHQ-9: Patient Health Questionnaire-9
RBF: radial basis function
RF: random forest
Project SHADO: Analyzing Social and Health Attributes through Daily Digital Observation
SVM: support vector machine
XGBoost: extreme gradient boosting
