# Supplementary material for: Using Passive Smartphone Sensing for Improved Risk Stratification of Patients With Depression and Diabetes: Cross-Sectional Observational Study
Source: JMIR Mhealth Uhealth. 2019 Jan 29;7(1):e11041. doi: 10.2196/11041 (PMC6371066; doi:10.2196/11041)
Supplement: Multimedia Appendix 1 [file mhealth_v7i1e11041_app1.pdf]

PATIENT HEALTH QUESTIONNAIRE-9 (PHQ-9)

**Over the <u>last 2 weeks</u>, how often have you been bothered by any of the following problems?** *(Use "✔" to indicate your answer)*	**Not at all**	**Several days**	**More than half the days**	**Nearly every day**
**1.** Little interest or pleasure in doing things	0	1	2	3
**2.** Feeling down, depressed, or hopeless	0	1	2	3
**3.** Trouble falling or staying asleep, or sleeping too much	0	1	2	3
**4.** Feeling tired or having little energy	0	1	2	3
**5.** Poor appetite or overeating	0	1	2	3
**6.** Feeling bad about yourself — or that you are a failure or have let yourself or your family down	0	1	2	3
**7.** Trouble concentrating on things, such as reading the newspaper or watching television	0	1	2	3
**8.** Moving or speaking so slowly that other people could have noticed? Or the opposite — being so fidgety or restless that you have been moving around a lot more than usual	0	1	2	3
**9.** Thoughts that you would be better off dead or of hurting yourself in some way	0	1	2	3

FOR OFFICE CODING ___0___ + ______ + ______ + ______

=Total Score: ______

If you checked off <u>any</u> problems, how <u>difficult</u> have these problems made it for you to do your work, take care of things at home, or get along with other people?

**Not difficult at all**	**Somewhat difficult**	**Very difficult**	**Extremely difficult**
☐	☐	☐	☐

Developed by Drs. Robert L. Spitzer, Janet B.W. Williams, Kurt Kroenke and colleagues, with an educational grant from Pfizer Inc. No permission required to reproduce, translate, display or distribute.
