# Supplementary material for: Using Passive Smartphone Sensing for Improved Risk Stratification of Patients With Depression and Diabetes: Cross-Sectional Observational Study
Source: JMIR Mhealth Uhealth. 2019 Jan 29;7(1):e11041. doi: 10.2196/11041 (PMC6371066; doi:10.2196/11041)
Supplement: Multimedia Appendix 2 [file mhealth_v7i1e11041_app2.pdf]

रुग्ण आरोग्य प्रश्नावली - 9
(PHQ-9)

**मागील 2 आठवड्यांच्या काळात, आपल्याला खालील पैकी कोणत्याही समस्येमुळे कितीवेळा त्रास झाला आहे ?** *(आपले उत्तर '✓' अशी खूण करून द्या)*	अजिबात नाही	अनेक दिवस	अर्ध्याहून अधिक दिवस	जवळपास प्रत्येक दिवशी
**1.** गोष्टी करण्यात थोडीशी रुचि किंवा आनंद	0	1	2	3
**2.** हताश, उद्वीग्नता, किंवा निराश वाटणे	0	1	2	3
**3.** झोप लागण्यात किंवा झोपलेले राहण्यात समस्या, किंवा खूप झोप येणे	0	1	2	3
**4.** थकलेले किंवा थोडी ऊर्जा असल्याचे वाटले	0	1	2	3
**5.** भूक मंदावणे किंवा अति खाणे	0	1	2	3
**6.** स्वतःबद्दल वाईट वाटणे — किंवा आपण अपयशी आहोत किंवा आपण स्वतःचा किंवा आपल्या कुटुंबाचा अपेक्षाभंग केला आहे असे वाटणे	0	1	2	3
**7.** वर्तमानपत्र वाचणे किंवा टेलिव्हिजन पाहणे यासारख्या गोष्टींवर लक्ष एकाग्र करण्यास त्रास होणे	0	1	2	3
**8.** हालचाल किंवा बोलणे इतके संथ होते की इतर लोकांच्या लक्षात येणे? किंवा याच्या उलट — इतके चिंताक्रांत किंवा अस्वस्थ होणे की आपण सामान्यपेक्षा बरेच अधिक इकडे-तिकडे फिरत आहात	0	1	2	3
**9.** आपण मेलो असतो तर चांगले झाले असते किंवा स्वतःला काही प्रकाराने जखमी करुन घेण्याचे विचार	0	1	2	3

FOR OFFICE CODING __0__ + ______ + ______ + ______

=Total Score: ______

आपण कोणत्याही समस्येवर खूण केल असेल तर, आपले काम करणे, घरी वस्तुंची काळजी घेणे, किंवा इतर लोकांसोबत वावरणे यामधे या समस्यांनी आपल्याला किती अवघड झाले ?

अजिबात अवघड झाले नाही	थोडेफार अवघड	खूप अवघड	अत्यंत अवघड
☐	☐	☐	☐

Drs. Robert L. Spitzer, Janet B.W. Williams, Kurt Kroenke आणि सहकारी यांच्याद्वारे Pfizer Inc. यांच्याकडून प्राप्त शैक्षणिक अनुदानातून विकसित. प्रतिलिपी, भाषांतर,प्रकाशन करणे आणि वाटप ह्यासाठी परवानगीची आवश्यकता नाहीं.
