# Supplementary figures and images for: Using Passive Smartphone Sensing for Improved Risk Stratification of Patients With Depression and Diabetes: Cross-Sectional Observational Study
Source: JMIR Mhealth Uhealth. 2019 Jan 29;7(1):e11041. doi: 10.2196/11041 (PMC6371066; doi:10.2196/11041)

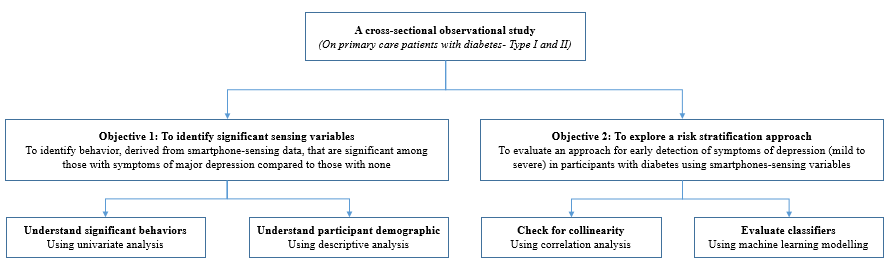

Supplement: Multimedia Appendix 3 [file mhealth_v7i1e11041_app3.png]

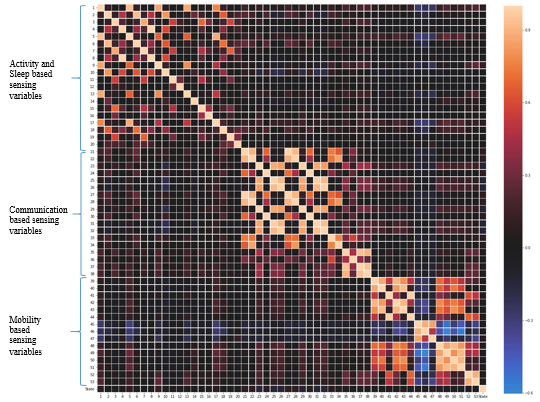

Supplement: Multimedia Appendix 6 [file mhealth_v7i1e11041_app6.png]
