# Supplementary material for: Using Passive Smartphone Sensing for Improved Risk Stratification of Patients With Depression and Diabetes: Cross-Sectional Observational Study
Source: JMIR Mhealth Uhealth. 2019 Jan 29;7(1):e11041. doi: 10.2196/11041 (PMC6371066; doi:10.2196/11041)
Supplement: Multimedia Appendix 4 [file mhealth_v7i1e11041_app4.pdf]

Multimedia Appendix 4: Derived Smartphone Sensing Variables

Ref No.	Feature	Derived smartphone-sensing Variable	Description
0	Symptoms of Depression State	PHQ-9 states (Dependent variable)	Binary (Depressed or Not Depressed).<br><br>Based on self-reported symptoms of depression score on PHQ-9
1	Activity and Sleep based Sensing	Active_Total	Total number of “active[a]” over a day polled every 2 minutes
2		Screen_On	Total number of ”Screen on[b]” over a day polled every 2 minutes
3		Rel_Grav_SD	Standard deviation of “relgrav[c]” over a day polled every 2 minutes
4		Light_SD	Standard deviation of “Light[d]” over a day polled every 2 minutes
5		Active_Day	Total number of “active” over the day-time[e] for the user polled every 2 minutes
6		Day_Screen_On	Total number of ”Screen on” over the day-time polled every 2 minutes
7		Day_Rel_Grav_SD	Standard deviation of “relgrav” over the day-time polled every 2 minutes
8		Day_Light_SD	Standard deviation of “Light” over the day-time polled every 2 minutes
9		Active_Eve	Total number of “active” over the evening[f] for the user polled every 2 minutes
10		Screen_On_Eve	Total number of ”Screen on” over the evening polled every 2 minutes
11		Rel_Grav_SD_Eve	Standard deviation of “relgrav” over the evening polled every 2 minutes
12		Light_SD_Eve	Standard deviation of “Light” over the evening polled every 2 minutes

Ref No.	Feature	Derived smartphone-sensing Variable	Description
13		Active_Night	Total number of “active” over the night-time[g] for the user pollled every 2 minutes
14		Screen_On_Night	Total number of ”Screen on” over the night-time sampled every 2 minutes
15		Rel_Grav_SD_Night	Standard deviation of “relgrav” over the night-time sampled every 2 minutes
16		Light_SD_Night	Standard deviation of “Light” over the night-time sampled every 2 minutes
17		Active_AM	Total number of “active” over the morning hours[h] for the user polled every 2 minutes
18		Screen_On_AM	Total number of ”Screen on” over the morning hours polled every 2 minutes
19		Rel_Grav_SD_AM	Standard deviation of “relgrav” over the morning hours polled every 2 minutes
20		Light_SD_AM	Standard deviation of “Light” over the morning hours polled every 2 minutes
21	Communication based sensing	variables.calls.data.total_calls	Total number of calls (made and received) over a day
22		variables.calls.data.missed_calls	Total number of missed calls over a day
23		variables.calls.data.total_people_called	Total number of unique people called (made and received) over a day
24		variables.calls.data.total_call_duration	Total duration (mins) of calls (made and received) over a day
25		variables.calls.data.call_count_entropy	Entropy[i] of call count with respect to distinct people called over a day
26		variables.calls.data.call_duration_entropy	Entropy of call duration with respect to distinct people called over a day
27		variables.peak_calls.data.total_calls	Total number of calls (made and received) over peak hours[j]

Ref No.	Feature	Derived smartphone-sensing Variable	Description
28		variables.peak_calls.data.missed_calls	Total number of missed calls over peak hours
29		variables.peak_calls.data.total_people_called	Total number of unique people called over peak hours
30		variables.peak_calls.data.total_call_duration	Total duration (mins) of calls (made + received) over peak hours
31		variables.peak_calls.data.call_count_entropy	Entropy of call count with respect to distinct people called over peak hours
32		variables.peak_calls.data.call_duration_entropy	Entropy of call duration with respect to distinct people called over peak hours
33		variables.off_peak_calls.data.total_calls	Total number of calls (made and received) over off-peak hours[k]
34		variables.off_peak_calls.data.missed_calls	Total number of missed calls over off-peak hours
35		variables.off_peak_calls.data.total_people_called	Total number of unique people called over off-peak hours
36		variables.off_peak_calls.data.total_call_duration	Total duration (mins) of calls (made and received) over off-peak hours
37		variables.off_peak_calls.data.call_count_entropy	Entropy of call count with respect to distinct people called over off-peak hours
38		variables.off_peak_calls.data.call_duration_entropy	Entropy of call duration with respect to distinct people called over off-peak hours
39	Mobility based sensing	variables.mobility_radius.data.total	The maximum distance (meters) between any two geographic coordinates visited by a user over a day
40		variables.mobility_radius.data.peak	The maximum distance (meters) between any two geographic coordinates visited by a user over peak hours
41		variables.mobility_radius	The maximum distance (meters) between

Ref No.	Feature	Derived smartphone-sensing Variable	Description
		.data.off_peak	any two geographic coordinates visited by a user over off-peak hours
42		variables.distance_travelled.data	The sum of distance (meters) between every two consecutive geographic coordinates visited by a user over a day
43		variables.peak_distance_travelled.data	The sum of distance (meters) between every two consecutive geographic coordinates visited by a user over peak hours
44		variables.off_peak_distance_travelled.data	The sum of distance (meters) between every two consecutive geographic coordinates visited by a user over off-peak hours
45		variables.time_spent_at_home.data.total	Total time (mins) spent at "home location[l]" by the user over a day
46		variables.time_spent_at_home.data.peak	Total time (mins) spent at "home location" by the user over peak hours
47		variables.time_spent_at_home.data.off_peak	Total time (mins) spent at "home location" by the user over off-peak hours
48		variables.distinct_locations.number_of_locations	Total number of "distinct locations[m]" visited by a user over a day
49		variables.distinct_locations.entropy	Entropy of number geographic coordinates detected for the user with respect to distinct locations over a day
50		variables.peak_distinct_locations.number_of_locations	Total number of "distinct locations" visited by a user over peak hours
51		variables.peak_distinct_locations.entropy	Entropy of number geographic coordinates detected for the user with respect to distinct locations over peak hours
52		variables.off_peak_distinct_locations.number_of	Total number of "distinct locations" visited by a user over off-peak hours

Ref No.	Feature	Derived smartphone-sensing Variable	Description
		_locations	
53		variables.off_peak_distinct_locations.entropy	Entropy of number geographic coordinates detected for the user with respect to distinct locations over off-peak hours

[a] **Active:** Where Relgrav values that exceeds the stationary threshold range (0.8 to 1.2)
[b] **Screen On:** Where the user had their mobile screen switched on and unlocked.
[c] **Relgrav:** Relative gravity is a measure of acceleration experienced by the mobile device with reference to earth's gravity. This is calculated by collecting X-Y-Z axis acceleration from the device and then taking the ratio of the sum of squares of X, Y and Z axis values and square of gravity. A stationary device was considered to have relgrav ~1 (range: 0.8 to 1.2). While falling the ratio was considered <1 and while being lifted the ratio was considered >1
[d] **Light:** The total quantity of visible light in Lumens (SI unit) as detected by the light sensor in the device at a given instant.
[e] **Day-time hours:** 12:00 hours to 16:00 hours
[f] **Evening hours:** 17:00 hours to 23:59 hours
[g] **Night-time hours:** 00:00 hours to 6:00 hours
[h] **Morning hours:** 6:00 hours to 11:59 hours (AM)
[I] **Entropy:** is a measure of the uncertainty or unpredictability associated with a variable. It is measured as entropy of a variable with respect to x (where x is a variable with possible values x_i). Entropy= - $\sum_i (p(x_i) * \ln(p(x_i)))$ where p(x_i) is probability of the x_i th value

[j] **Peak hours:** 8 AM to 10 PM for the user
[k] **Off-Peak hours:** 10 PM to 8 AM for the user
[l] **Home Location:** Geographic coordinates estimated to be the user's home location in a 250 metres radius. This is obtained by clustering user's locations during night time over 14 days
[m] **Distinct Location:** A region of diameter 250 metres within which one or more geographic locations lie.
