# Supplementary material for: Using Passive Smartphone Sensing for Improved Risk Stratification of Patients With Depression and Diabetes: Cross-Sectional Observational Study
Source: JMIR Mhealth Uhealth. 2019 Jan 29;7(1):e11041. doi: 10.2196/11041 (PMC6371066; doi:10.2196/11041)
Supplement: Multimedia Appendix 5 [file mhealth_v7i1e11041_app5.pdf]

Multimedia Appendix 5: Measures taken to ensure sensing data integrity and accuracy

Data Integrity measures include:

- Several iterations of the sensing toolkit being done on the app to ensure that low-end devices with limited connectivity polled data periodically (every 2 minutes), and sent to the servers.
- In case of limited or no connectivity, data was polled and stored locally on the device. This was then batched and transmitted when internet became available. This ensured that even when a participant remained disconnected to the internet for days, the data was not lost.
- Workarounds were devised to ensure that the device operating system did not kill the data gathering, while still keeping a minimal footprint on the device resources in terms of battery, memory and data usage.

Data Accuracy/Validity measures include:

- Data like activity and distance travelled were periodically sampled by the study investigating team and patterns were confirmed with participants to ensure data collected was accurate.
- Different devices were tested under various conditions to compare and establish the accuracy of readings collected from the smartphone sensors. For example, we saw variations in relative gravity readings at very low values from the accelerometer for very low-cost devices versus higher-end devices, and this helped us create thresholds within which the data collected was accurate across multiple devices.
- To further minimize the impact of data accuracy, for many derived variables in the analysis dataset, we opted to use normalized or relative values rather than absolute readings. For example, we used standard deviation for appropriate sensor readings rather than mean or median values to normalize differences between different types of hardware.
- For variables related to location, the investigating team set a minimum threshold of accuracy for which participant data and context would be meaningful. Data polling was continued until this threshold was met, and then confirmed with participants.
